# Validation of the Work Observation Method By Activity Timing (WOMBAT) method of conducting time-motion observations in critical care settings: an observational study

**DOI:** 10.1186/1472-6947-11-32

**Published:** 2011-05-17

**Authors:** Mark A Ballermann, Nicola T Shaw, Damon C Mayes, RT Noel Gibney, Johanna I Westbrook

**Affiliations:** 1 Department of Family Medicine, University of Alberta, Edmonton, Alberta, Canada; 2 Health Informatics Institute, Room SH 500. Algoma University, 1520 Queen Street East, Sault Ste. Marie, Ontario, P6A 2G4, Canada; 3 Alberta Health Services, Edmonton, Alberta, Canada; 4 Division of Critical Care Medicine, Faculty of Medicine & Dentistry, University of Alberta, Edmonton, Alberta, Canada; 5 Centre for Health Systems and Safety Research, Australian Institute of Health Innovation, Faculty of Medicine, University of New South Wales, Sydney, Australia

## Abstract

**Background:**

Electronic documentation handling may facilitate information flows in health care settings to support better coordination of care among Health Care Providers (HCPs), but evidence is limited. Methods that accurately depict changes to the workflows of HCPs are needed to assess whether the introduction of a Critical Care clinical Information System (CCIS) to two Intensive Care Units (ICUs) represents a positive step for patient care. To evaluate a previously described method of quantifying amounts of time spent and interruptions encountered by HCPs working in two ICUs.

**Methods:**

Observers used PDAs running the Work Observation Method By Activity Timing (WOMBAT) software to record the tasks performed by HCPs in advance of the introduction of a Critical Care clinical Information System (CCIS) to quantify amounts of time spent on tasks and interruptions encountered by HCPs in ICUs.

**Results:**

We report the percentages of time spent on each task category, and the rates of interruptions observed for physicians, nurses, respiratory therapists, and unit clerks. Compared with previously published data from Australian hospital wards, interdisciplinary information sharing and communication in ICUs explain higher proportions of time spent on professional communication and documentation by nurses and physicians, as well as more frequent interruptions which are often followed by professional communication tasks.

**Conclusions:**

Critical care workloads include requirements for timely information sharing and communication and explain the differences we observed between the two datasets. The data presented here further validate the WOMBAT method, and support plans to compare workflows before and after the introduction of electronic documentation methods in ICUs.

## Background

Some of the most acute patients in hospital settings are treated in Intensive Care Units (ICUs) by specialized Health Care Providers (HCPs). HCPs use diverse information sources to prioritize their tasks and make decisions about patient care. These sources include other HCPs, bedside equipment, and laboratories located at some distance from the patient [1-3]. HCPs coordinate care over time and across HCP roles in a way that is consistent with patient needs, and thus strive for continuity of care [4]. Continuity of care is thought a crucial determinant of patient outcome, and depends on the timely availability of patient information or informational continuity. Patient charts contain critical information about patient status and care plans, and support medical decision making.

HCPs working in ICUs must manage many information sources to ensure that the documentation in each patient chart is correct, current, and complete. With paper charts in place, HCPs transcribe information from bedside equipment and laboratory reports. Transcribing information into patient charts may not be the best use of time for highly specialized and expensive HCPs. An electronic medical record designed for the ICU environment, a Critical Care clinical Information System (CCIS), can automate some transcription tasks and aid in informational continuity between HCPs [5]. The results of studies to date examining whether a CCIS will reduce time spent on documentation have been equivocal [6,7]. Moreover, high rates of failure have been reported for many electronic systems in healthcare, in which organizations have either adopted other systems or reverted to paper charts [8,9].

Evaluation methods of HCP work can be used before and after a system introduction to collect more objective data about its impact on ICU operations. Impacts on ICU operations will include impacts to the workflows of HCPs including physicians, nurses, and others. Observational and self-reporting techniques have been used to study work in hospitals and other settings [10,11]. A practical advantage of self report studies is the lower cost resulting from participants recording their own activities when they are prompted by a reminder device [10,12]. However, participants giving self reports may ignore the prompts during busy work periods, which can result in incomplete data [10,11,13]. In ICU environments, tasks performed during busy work periods may be particularly critical for patient outcomes and thus may be systemically neglected by work sampling methods. In comparison, observational techniques employ a researcher to observe and record the behaviours of interest, which significantly reduces concerns about incomplete data [10]. Others have used continuous observation to examine the overall time spent by health care providers (such as physicians) on tasks in hospital ward settings [14,15]. However, little is currently known about the proportions of time that HCPs spend on their various tasks while working in ICUs.

Furthermore, comparisons between ICUs and general hospital wards have shown that there are higher rates of adverse medical events in ICUs [16]. Interruptions and their associated shifts in cognitive focus may be a factor in medical error [17]. A clear picture of how HCPs working in ICUs manage interruptive communications is needed to better understand the relationship between interruptions and errors [17-19]. The CCIS may facilitate access to information that is crucial to decision making associated with patient care. This paper is part of a larger study that will examine whether the use of a CCIS represents a positive step for ICU patient care and for HCPs working in ICUs. The availability of information in stored patient charts may improve after the CCIS introduction, but this is presently not known. The capture of myriad data sources may be facilitated by the introduction of a CCIS, but valid comparisons will benefit from more complete descriptions of methods for quantifying workflow (including proportions of time spent on tasks, and interruptions encountered) of HCPs in ICUs. Initial descriptions of the larger CCIS study design have been reported [20,21], and other reports have compared pre- and post-CCIS data [22-25].

### Rationale

This paper provides part of a methodological foundation for a larger mixed-methods study assessing the impact of a CCIS. We are conducting a study investigating two academic tertiary-care ICU units that introduced a CCIS in early 2009. The CCIS implementation is likely to impact both the quality and availability of information in patient charts, and it is likely to also affect HCP work.

This paper documents the methodology used to observe ICU nurses, physicians, unit clerks and respiratory therapists working with paper charts. We recorded the amounts of time they spent on their tasks using Personal Digital Assistants (PDAs) running Work Observation Method By Activity Timing (WOMBAT) software. We assert that this observation method allows us to derive valid and reliable measures of amounts of time HCP spend on tasks and interruption rates when compared with previous data [13,15]. Previous studies using the WOMBAT software have described task data definitions for nurses and physicians working in general hospital settings [13,15]. We extend these task data definitions to quantify the time spent on tasks by respiratory therapists and unit clerks. We collected observational data at shift changes, which are times when maintaining continuity of care is challenging, and balanced our data collection across all days of the week and times of day to capture a representative picture of work in the ICUs. Finally, we show evidence suggesting that HCPs do not alter their activities based on the presence of an observer, an effect commonly referred to as the Hawthorne effect [26]. This evidence may help to address concerns that care providers alter their activities as a result of being observed.

### Objective

In this paper we compare our time-motion baseline results with those previously published to provide supporting evidence that the WOMBAT method provides valid results when quantifying amounts of time spent on different tasks and interruptions encountered by critical care providers. We discuss differences observed between the reported data and previously published results from Australian general hospital wards [13,15] and emergency wards [27], based on the nature of critical care.

## Methods

### Research setting

The University of Alberta Human Research Ethics Board (File #B-241107) and Northern Alberta Clinical Trials and Research Centre (File #6035) granted approval for this study prior to the commencement of data collection. We conducted our study in the Pediatric ICU (PICU) at the Stollery Children's Hospital and the General Systems ICU (GSICU) at the University of Alberta hospital in Edmonton, Alberta, Canada. The PICU has 17 beds. The GSICU has 30 beds, with 24 operational during the observations due to staff shortages. These are busy critical care units in academic tertiary referral hospitals. At the time of the observations, the units operated with paper charts in place, with internet-enabled computers at nursing stations and throughout the unit. Laboratory data are available through these computers. The ratio of nurses to patients is 1:1 in the PICU, and 1:1 70% of the time and 1:2 30% of the time in the GSICU, depending on patient acuity.

### Participants

Members of the staff were informed of our study through presentations given by members of the research team and by posters distributed around the units. Participants were then approached by members of the research team for their consent to be observed.

Of 215 nurses in permanent staff positions, 87 agreed to participate (40%) and 47 were observed. Of 35 attending physicians and fellows, 32 agreed to participate (91%) and 18 were observed. Fellows included physicians at their third year of post medical degree training or above, working on the unit in a full-time capacity. Of 72 respiratory therapists working on the units, 46 agreed to participate (64%) and 25 were observed. We obtained consent from 14 out of 16 unit clerks working on the units (88%), and 10 were observed. Observations were randomly selected from the participants working in the ICUs, so participants who were scheduled for fewer shifts would be less likely to be observed. Informed consent and demographic data (age, sex, time employed in ICU settings, number of shifts per month, and self-assessed familiarity with computers) were obtained from participants.

### Observers

Observers were trained for at least 12 hours before starting observations. During training sessions, observers were oriented to the PDA software, the work definitions, and the ICUs. Observers would then follow a HCP alongside an experienced observer, simultaneously scoring the same tasks. The experienced observers were nurses working on the PICU. Inter-rater reliability scores were calculated for total time spent and numbers of tasks scored. Values of 85% or higher were obtained between the observers before trainees conducted their own observations.

### Work definitions

Observers carried a list of work definitions to assist in classifying tasks they observed into one of the categories in the PDA. These work definitions were initially provided by Westbrook and colleagues [15]. The work definitions were further refined to include tasks specific to the observed units, and tasks specific to the respiratory therapists and unit clerks. The complete work definitions for the nurses, physicians and respiratory therapists are provided in Table 1. Unit clerks collect and disseminate a great deal of information within the units. Separate work definitions were created for them as their tasks were fairly different from other HCP roles. The work definitions for unit clerks are provided in Table 2.

**Table 1:** Physician, Nurse, and Respiratory Therapist work definitions

Category	Includes	Excludes
**Direct Care** Any activity directly related to patient care.	Admitting a patient Examining/reviewing a patient Performing medical procedures Assisting other staff with procedure Escorting a patient Communicating with patient/relative Taking a history	Medication related activities Documenting Reviewing documentation/results Planning care Communicating with staff member

Note: All communication with patient/relative is defined as direct patient care. When the participant is discussing medications with a patient (e.g. as part of history taking and review), this is defined as a medication related event.		

**Indirect Care** Any activity indirectly related to patient care.	Reading & reviewing documents Planning care and ordering tests, diet etc. Running blood gases Retrieving information (from temporary or perm record, or computer) Checking results Washing hands Gathering & returning equipment Cleaning up after a procedure Watching monitors Find medical record-drop down Find x-ray/scan-drop down	Medication related activities Documenting in patient notes Communicating with staff member Communicating with patient/relative

Note: Monitors, ventilators, and other electronic patient care equipment should be coded as 'computer'.		

**Medication** Any activity that relates to medication for a particular patient.		

Medication: Find Order	Looking for medication charts	Looking for notes in general

Medication: Prescribe Drug	Writing up a new order Changing orders Receiving or requesting a verbal order Giving a verbal order Writing discharge scripts Obtaining drug authority numbers	Re-writing orders (e.g. legibility issues, needs signatures, etc.) Following a request for clarification (see Clarify)

Medication: Transcribe Order	Copying med orders from one medication chart to new one (e.g. for end of week continuing meds) Transcribing verbal order	Re-writing orders (e.g. legibility issues, needs signatures, etc.) following a request for clarification (see Clarify)

Medication: Prep Drug *Activity around drug preparation & clean-up*	Reading medication order to select drug Finding drug/or reconstitution fluid/or selection of appropriate equipment for preparation and administration Locating drug keys Preparing the drug (e.g. reconstitution, drawing up solution, crushing tablets, preparing nebulizers) Commencement and removal of IV infusions, cannulation, rearranging and disconnecting tubing for the purpose of drug administration Returning/disposing equipment &/or drug following administration S4/S8 counting & drug register entry for individual patients Checking the drug with other staff (when the participant who is being observed is responsible for the drug) Faxing/scanning order to pharmacy Re-ordering/re-filling drugs	

Medication: Clarify *Action taken when:* *Asked to clarify an order that has been previously written, or* *asking another doctor to clarify an order; or Seeking drug information for clarification (e.g. when prescribing)*	Re-writing a drug order due to illegibility or legal reasons (or asking a doctor to re-write) Checking the particular drug details in Mims or other source asking another health professional about the drug order Clarifying with the patient about the drug order	Asking another nurse or doctor to check a drug that the observed participant wants to give (see Prep drug)

Medication: Check Drug *Checking with & co-signing of a nurse's or another doctor's medication*	Witnessing any other medication for another health professional (e.g. IV a/b checks) Checking ID band &/or order &/or label	Asking another nurse or doctor to check a drug that the participant wants to give (see Prep drug)

Medication: Administering/Charting *Giving medication to a patient/recording drug administration details*	Any patient preparation (e.g. sitting patient up so they can swallow medication, etc.) Reviewing &/or taking &/or documenting vital signs as part of the protocol before giving the medication (e.g. pulse prior to digoxin) Checking &/or adjusting IV admin throughout the administration process (e.g. returning to the pt & checking tubing, drip rate, etc.) Documenting on medication order notes Silencing IV pumps	Co-signing (see check-drug) Drug register (see Prep drug or Check drug)

Medication: Discuss *Talking about a drug with another health professional &/or patient/relative*	Choice of drug &/or dosage Side effects Discharge education Efficacy Administration protocols	Clarification of an order (see Clarify) Requests for re-writing of order due to illegibility (see Clarify)

Medication: Review	Looking over drug orders as part of planning care	

**Document** Any recording of patient information on paper or computer.	Writing on temporary record (e.g. own list) Writing in patients' notes Getting physicians to sign-off on non-medication orders Discharge summaries-drop down	Medication chart documentation

**Professional Communication** Any work-related discussion with another staff member.	Requesting medical or nursing consult or review Planning care with any health professional Handover/parts of a ward round	Medication related discussion Communication with patient/relative

**Administrative** Any administrative activity that is not related to direct or indirect individual patient care. Also includes activities that relate to the running of the unit in general (but aren't related to direct or indirect patient care).	Duty rosters Employment issues Bed allocations Coordination of staff activities Staff meetings (not case or clinical meetings) Unit related Unit orders for stock	Handover Rounds

**In Transit** Work related movement between patients and between tasks.	Movement when the participant exits a patient room	Movement between patients in a shared room Movement within a single room

Note: When the participant arrives at another task or patient, "In Transit" ceases and the next appropriate category is chosen for that active task. e.g. participant leaves room to get equipment = in transit participant returns with equipment = indirect care		

**Supervision/Education** Active supervision or teaching of another staff member or student.	Attending education sessions (e.g. grand rounds)	

Note: When the participant is actively supervising, "supervision" is selected and all tasks normally undertaken by the participant are added under "multi".		

**Social** Any social or personal activity or discussion.	Personal phone calls, tea & personal breaks Bathroom breaks Reading books/magazines	

**Pager** Whenever the participant's pager alerts, pager is to be entered as an interruption.	Reading pager Returning call	Calling/having paged other healthcare providers (See Professional Communication)

**Table 2:** Unit clerk work definitions

Category	Includes	Excludes
**Direct Care** Any activity directly related to patient care. For unit clerks, direct patient care will typically only be entered when s/he talks to the patient's relatives.	Communicating with patient/relative	Ordering results Communicating with staff member

Note: All communication with patient/relative is defined as direct patient care.		

**Indirect Care** Any activity indirectly related to patient care.	Reading & reviewing documents directly related to a particular patient Organizing, ordering, and entering tests, diet, x-rays, blood products, etc. (includes ordering via computer) Retrieving information (from temporary or perm record, or computer) Check for results Washing hands Gathering & returning equipment	Medication related activities Documenting in patient notes Communicating with staff member Communicating with patient/relative

**Document** Any recording of patient information on paper or computer.	Writing on temporary record (e.g. own list) Registering patients (entering data into computer or onto paper chart)	Ordering tests, etc.

**Professional Communication** Any work-related discussion with another staff member.	Requesting medical or nursing consult or review Handover/parts of a ward round Receiving any request (e.g. order test, page physician)	Medication related discussion Communication with patient/relative

**Administrative** Any administrative activity that is not related to direct or indirect individual patient care. Also includes activities that relate to the running of the unit in general (but aren't related to direct or indirect patient care).	Answering visitor's phone Answering phone calls Duty rosters Employment issues Coordination of staff activities Staff meetings (not case or clinical meetings) Unit related Entering x-ray Paper work (e.g. organizing lab results to deliver)	Handover Rounds Making a phone call to someone that you know is professional

**In Transit** Work related movement.	Movement when the participant exits a patient room Movement between bedsides	Movement within a single room (e.g. if patient is in isolation room)

Note: When the participant arrives at another task or patient, "In Transit" ceases and the next appropriate category is chosen for that active task. e.g. participant leaves room to get equipment = in transit participant returns with equipment = indirect care		

**Supervision/Education** Active supervision or teaching of another staff member or student.	Attending education sessions (e.g. grand rounds)	

Note: When the participant is actively supervising, "supervision" is selected and all tasks normally undertaken by the participant are added under "multitasking".		

**Social** Any social or personal activity or discussion.	Personal phone calls, tea & personal breaks Bathroom breaks Reading books/magazines	

**Pager** Whenever the participant pages a physician, fellow, or another staff member.		

### Time motion observation tool

Observers carried Hewlett-Packard iPAQ hx2490 Personal Digital Assistants (PDAs) running the Work Observation Method By Activity Timing (WOMBAT) software [15]. Observers scored the start of tasks by selecting the task category (what was done), the people who were present (who the task was done with), and any information tools used and pressing the 'enter' button. The time that the enter button was pressed was then recorded as the start time for that task. Subsequent tasks could be scored by selecting new information and pressing enter. The end time of the previous task and the start time of the new task were both entered into the database automatically.

Participants were frequently observed performing more than one task simultaneously. The WOMBAT program allowed observers to score multiple simultaneous tasks using the multitasking function. New tasks could be added to those that were already underway if observers pressed the 'add' button prior to pressing the 'enter' button. The presence of different 'tabs' allowed the observer to see the tasks that were being scored at the current time. If one or more tasks ended, individual tasks could be scored as stopped using an 'end multi' button. The software required that at least one task be scored at all times.

If any external factor appeared to cause a HCP to stop performing one or more tasks, and start another task, an 'interruption' was recorded. Observers entered the task information for the task that was started and pressed the 'interrupt' button. The first task was moved into a background tab as a 'pending' task that the care provider may or may not return to. Observers could remove pending tasks if it appeared that the care provider would not come back to them.

Data were extracted into Excel spreadsheets via a laptop computer. Proportions of time spent on the different task categories and the rates of interruption were calculated for each observation. Interruptions were characterized by the task the participant initiated after the interruption was scored.

### Observations

Observations were carried out for 90 minutes with no advance warning to the participant. The 90 minute time limit is based on the notion that this may represent an upper limit to the length of time in which observers may be capable of recording all the tasks they observe. Equal numbers of observations were performed in 4 conditions: mid-shift during the day (07:00-19:00), mid-shift at night (19:00-07:00), during the morning shift change (06:30-08:00), and evening shift change (18:30-20:00). Observations were also balanced between 4 day types: midweek, weekend, Mondays, and Fridays. Workload is likely to vary with the day of the week as the number of operating rooms in use impacts the mix of admitted ICU patients. Mondays and Fridays would likely have more admission and discharge activity, respectively. These factors would impact the workload encountered by participants. Observers kept field notes for recording contextual information, such as their impression of how busy the unit was, whether the unit was short-staffed, and whether there were students present on the unit. Observers maintained a following distance of approximately 3 metres.

Observations were suspended when participants left the unit or went on a break. The goal of this study was to collect observational data in the unit environment where the CCIS would be put into place, rather than tasks performed elsewhere. The observations in the PICU were carried out between September and November, 2008. The observations in GSICU were carried out between January and February 2009. We performed 62 hours of nurse observations, 58 hours of physician observations, 55 hours of respiratory therapist observations and 57 hours of unit clerk observations.

### Statistical analysis

Although participants were assured that their personal work was not being evaluated when informed consent was obtained, the possibility exists that participants may not recollect that reassurance. As such, participants may have an incentive to avoid tasks that would be scored as 'social' time. Participants may habituate to the presence of an observer, so differing proportions of social time during the early parts of an observation compared with the overall proportions may indicate a phenomenon known as participant reactivity or the Hawthorne effect [26]. Proportions of time spent on social tasks in the first minute, first five minutes, and first ten minutes were compared to the similar proportions from the entire observation with Mann-Whitney U tests. These tests were completed to examine whether HCPs tended to avoid socializing when an observation started, but eventually habituated to the presence of an observer.

## Results

We observed 14,928 separate tasks across all HCP roles. The mean time spent per task was 78 seconds, with a median task time of 34 seconds. The maximum time spent on one task was 39 minutes when a respiratory therapist attended a meeting. ICU physicians, nurses, respiratory therapists and unit clerks were observed for 58 hours, 62, 55, and 57 hours respectively.

### Percentages of time spent on tasks

**Figure 1:**
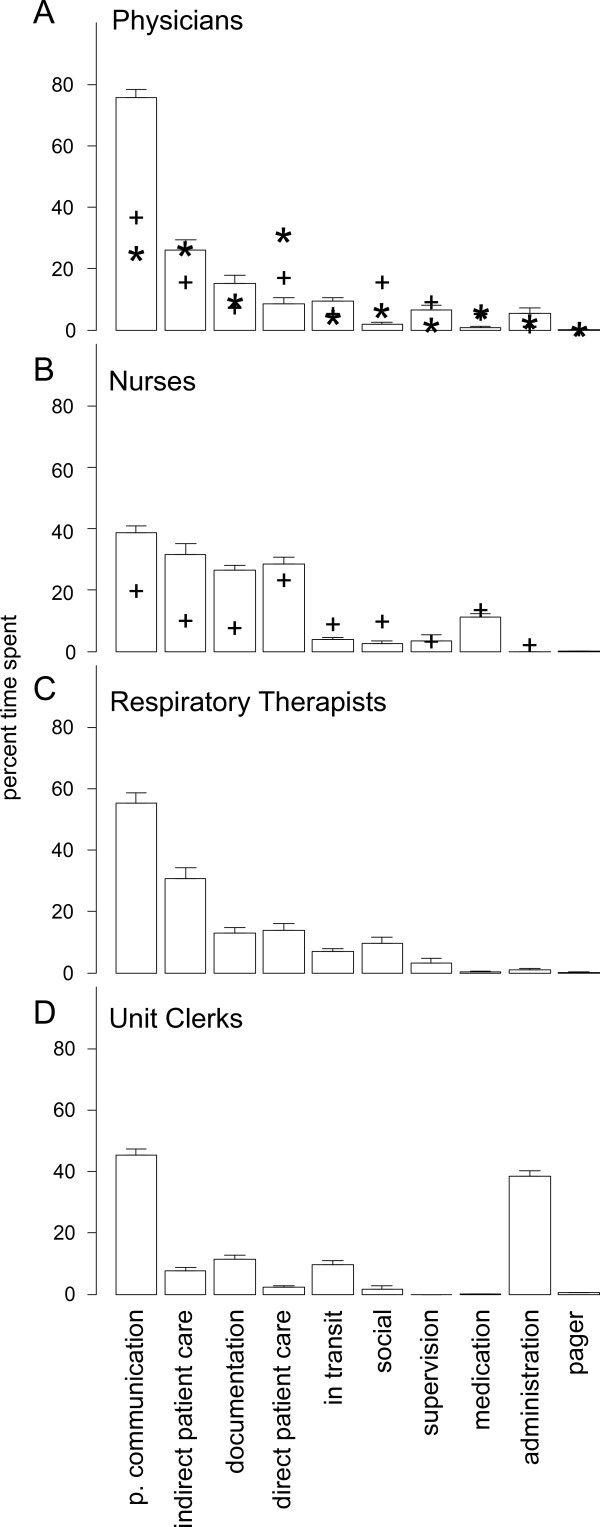
**Time percentages spent performing different tasks by critical care providers**. Values represent means and error bars represent 95% confidence intervals. Plus signs (+) in A represent values for percentages of time spent by physicians in general hospital wards on tasks taken from [13], and in B corresponding values for nurses were adapted from [15]. Asterisks in A represent percentage of time spent by physicians working in EDs, adapted from [27]. P. Communication = Professional Communication.

Total percentages of time spent on task categories by physicians, nurses, respiratory therapists, and unit clerks, were calculated and presented in Figure 1. Comparable time percentages from two Australian datasets [13,27] of observations of physician work (Figure 1A) and one dataset [15] of nurse work (Figure 1B) were superimposed to facilitate comparisons. Critical care providers spent large percentages of time performing more than one task at a time, or multitasking, a finding that has been previously reported [23]. We found that the WOMBAT method yields data that are generally consistent with the Australian datasets [13,15,27], with only minor differences. Physicians and nurses in the Australian datasets spent less time on professional communication tasks than ICU nurses and physicians. For nurses, these findings may be accounted for by ICU patient to nurse ratios being closer to 1:1, compared with much higher ratios on general hospital wards. Due to the highly acute nature of ICU patients, we frequently observed nurses working together to perform patient care tasks. For example, several nurses may be required to bathe a heavier sedated patient. High levels of teamwork in ICUs likely account for the greater proportions of professional communication among nurses. Similarly, ICU physicians frequently participate in morning rounds, afternoon "sign-out" rounds, and nightly rounds as part of a multidisciplinary team, including nurses, respiratory therapists, resident physicians, nutritionists, pharmacists, and social workers. Additionally, consulting physicians often arrive to speak with the attending physicians about patients in the ICU. This team-based approach to providing health care in the ICU likely accounts for the very high values we observed in professional communication.

Nurses spent greater amounts of time on documentation tasks in ICUs, a difference from the Australian general ward dataset [15]. This may be partly attributable to the greater requirements for regular monitoring and documentation of ICU patients [28]. Rapidly changing clinical conditions of ICU patients may necessitate more frequent and detailed documentation than is the case for patients on general hospital wards. The myriad information sources in the ICU, including bedside telemetry, lab results, observations, and procedures performed by HCPs all need to be documented from a medico-legal standpoint as well as to inform care providers of the current and possibly rapidly changing state of the patient.

### Interruptions

**Figure 2:**
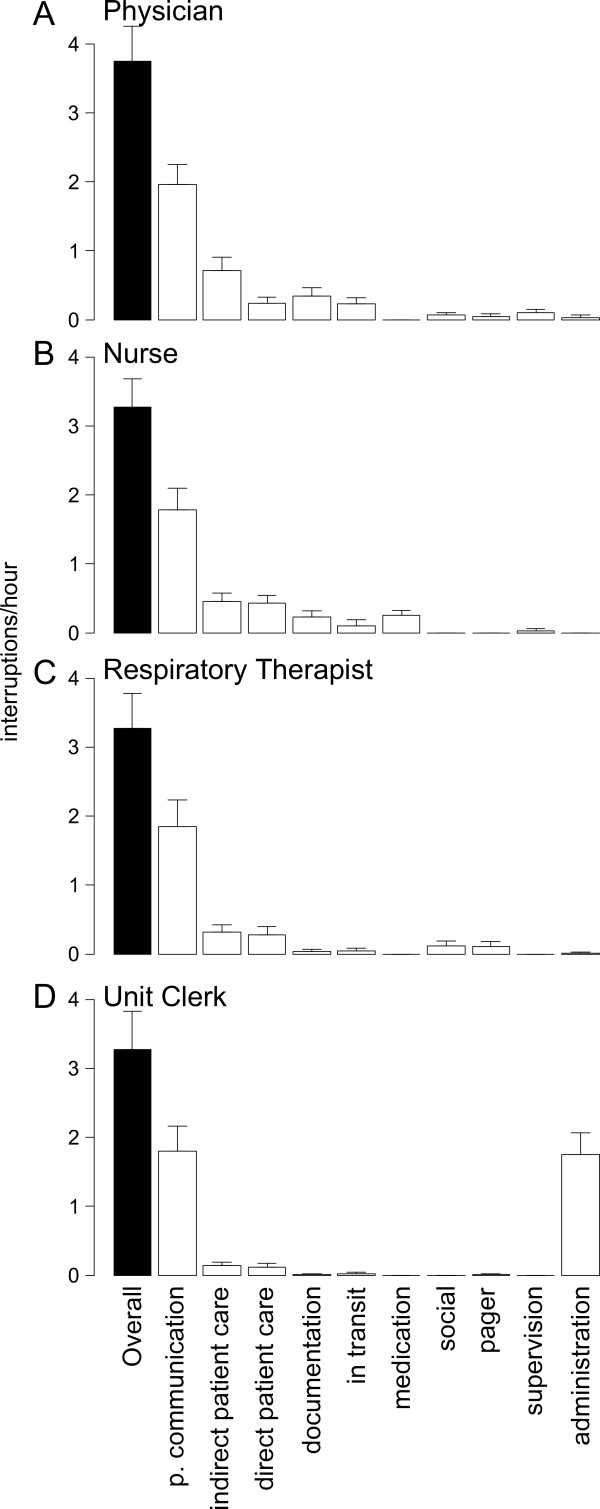
**Interruption rates for critical care providers**. Values represent mean interruption rates for 90 minute observations. Error bars represent 95% confidence intervals. Black bars represent overall rates of interruption. White bars represent the rates of interruptions where HCPs where the secondary (interrupting) task was the task named at the bottom. P. Communication = Professional Communication.

We recorded mean interruption rates for ICU physicians of 3.8 times per hour (once every 15.8 minutes on average; Figure 2A). Nurses were interrupted 3.3 times per hour, an average of an interruption every 18.3 minutes (Figure 2B). Respiratory therapists were interrupted 3.5 times per hour, on average, corresponding to an average time between interruptions of 17 minutes (Figure 2C). Unit clerks were interrupted 4.4 times per hour on average, corresponding to an interruption every 13.8 minutes (Figure 2D). For all roles observed, the tasks initiated after an interruption were most often professional communication tasks. For physicians, nurses, respiratory therapists, and unit clerks, interruptions where these tasks were initiated occurred at rates of 2.0 interruptions/hour (or hr^-1^), 1.8 hr^-1^, 1.9 hr^-1^, and 1.8 hr^-1^, respectively. HCPs may need to collect and disseminate information in a timelier manner in ICUs than on other units. As a consequence, interruptions may be more frequent in the ICU setting than on general hospital wards [13,15]. As a part of our larger study we have examined HCP perceptions of interruptions through interviews and focus groups. HCPs of all types reported that interruptions are pervasive in ICUs [25]. The reports of more frequent interruptions are confirmed by the observational data provided here. ICU physicians were interrupted 3.8 times per hour whereas comparable physicians on hospital wards experienced 2.9 interruptions per hour [13]. Interruptions in EDs have also been studied using the WOMBAT method, where ED physicians encountered 6.6 interruptions/hour, which is comparable to other observations of ED physicians [29,30]. We find that ICU physicians were interrupted less often than ED physicians, which is axiomatic.

### Participant reactivity

**Figure 3:**
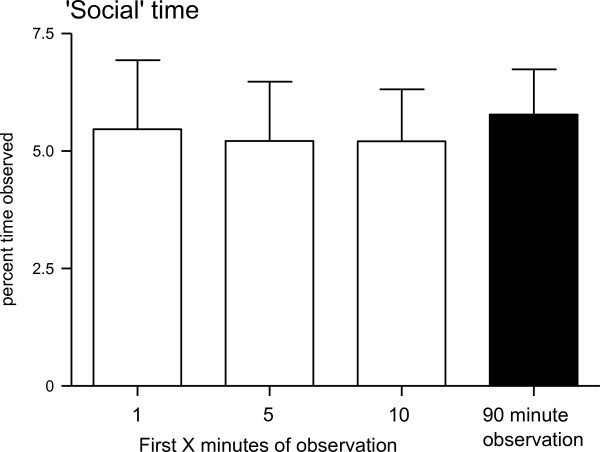
**Percentages of time spent on social tasks**. Percentages of observed time spent on social time were calculated across all health care provider roles for the first minute, 5 minutes, 10 minutes and entire 90 minute observation. Values represent mean percentages of time spent for each sampling period. Error bars represent 95% confidence intervals.

If participants change their behaviour as a result of being observed [26], such changes should be most evident at the start of an observation when participants first learn that they will be observed for the next 90 minutes. As participants habituate to the presence of the observer, we might expect Hawthorne-like effects to lessen. As an approach to exploring whether Hawthorne-like effects exist within our data, we examined amounts of time recorded as 'social' tasks. All participants were assured that their personal performance would not be evaluated, yet some individuals expressed some scepticism to the assertion that they were not being personally evaluated. As such, individual participants may have felt uncomfortable engaging in social tasks in the presence of observers. To examine the possibility of participants altering their behaviour due to the presence of an observer, the percentages of time spent on social activities were calculated per session for the first minute, 5 minutes, 10 minutes, and for the entire 90 minute observation (Figure 3). There were no significant changes between the mean proportions of time spent on activities scored as 'social' for the early periods of the observation compared to the entire observation, a result that may be inconsistent with participants altering their behaviour as a result of being observed.

## Discussion

We report the percentages of time that HCPs working in two Canadian ICUs spent on different categories of tasks, and the interruptions encountered using a method previously applied to quantify the work of physicians [13,27] and nurses [15] in Australian hospitals. Based on the results reported here, we posit that this method is a valid approach for collecting time and motion data in health care settings to assess the amounts of time spent on different tasks.

We make strong comparisons between the Australian datasets [13,15,27] and our own data from ICUs as we used nearly identical data definitions, with minor changes to account for tasks specific to the two units participating in our study and those tasks specific to the respiratory therapist and unit clerk roles, which were not assessed previously. Where our findings vary from those in the literature, they make theoretical sense based on the ICU observational setting, where complex patients are managed by teams of specialized care providers. We applied this methodology to observe respiratory therapists and unit clerks, to obtain a more complete picture of how critical care changes after a CCIS introduction. We conducted observations during nurse, respiratory therapist, and unit clerk shift changes, to examine workflows during times when informational continuity was challenged [31]. The results demonstrate the amounts of time critical care providers spend dealing with information to address the needs of highly complex and acute patients and provide a basis to assess the impacts of a CCIS on critical care. Future studies will examine whether the introduction of a CCIS facilitates the access to information and the completion of documentation tasks associated with patient care.

Importantly, we show for the first time the proportions of time spent by respiratory therapists and unit clerks in the critical care setting. We applied the WOMBAT method to observe respiratory therapists and unit clerks, and reported the amounts of time spent by HCPs in each role on the different task categories. We refined the work definitions to include the tasks performed by these HCPs. Much like the physicians and nurses in the ICUs, respiratory therapists spent high percentages of time performing professional communication tasks. Indirect and direct patient care and documentation were additional major categories of tasks. Respiratory therapists often care for multiple patients on the units, depending on the patient load. Due to the physical layout of the GSICU, respiratory therapists on GSICU spent more time 'In transit' than any other HCP role we observed. These findings objectively show the quantities of time spent on each task category, and illustrate the nature of workflow of respiratory therapists on the two units.

Unit clerks spent a significant proportion of their time on professional communication and administration tasks. The unit clerk is a central focus of information flow throughout the unit. Unit clerks assist by providing information that is critical to patient care by communicating with other HCPs to manage the timing of family visits and ensure the delivery of effective patient care.

ICU care providers encounter interruptions at rates that are between those of care providers working in EDs and general hospital wards. Of these interruptions, half are followed by professional communication tasks. As an association between interruptions and medication errors has been shown [32], understanding the reasons why interruptions are pervasive in certain environments is a prerequisite to improving patient safety. As many interruptions in ICUs are related to the management of information, the introduction of a CCIS may result in changes to the rates and types of interruptions encountered by critical care providers. Future studies will focus on the rates and types of interruptions and how they are both perceived and managed by HCPs on ICUs [24,25].

### Participant reactivity

One concern that is frequently raised about observational studies concerns the phenomenon of participant reactivity, when a participant may change their behaviour as a result of being aware that they are being studied [26]. Also called the Hawthorne effect, its original appearance has been called into question by a more recent careful re-examination of the original data [33]. We examined the proportions of time spent on 'social' tasks during the first minute, 5 minutes, and 10 minutes across observations for all roles, based on the likelihood that if participants changed their work significantly due to being observed, a likely change may be avoiding actions that would be scored as 'social', especially at the outset of an observation. We found no significant differences between the proportions of time spent on 'social' activities during any of these time periods and the entire observation periods, which is not consistent with a Hawthorne-like effect. This interpretation of the data depends on the assumption that Hawthorne-like effects would extinguish as the observation continues, but if this assumption is incorrect, the results reported would fail to identify a Hawthorne-like effect. However, various observational studies of clinicians *in situ *have suggested that the extent of behaviour change is minimal [10,34,35]. Although it is difficult to completely rule out Hawthorne-like effects as factors in general, these findings are inconsistent with the Hawthorne effect being a factor in our data. It is important to note that the nature of the critical care environment requires staff to quickly adjust to the presence of many different members of the health care team. HCPs on ICUs within teaching hospitals, in particular, may be less likely to demonstrate Hawthorne-like effects because of their experience in performing tasks in the presence of many observers. Finally, our observers included ICU nurses, some of whom were familiar to the participants.

### Strengths and limitations

This study provides valuable information about the tasks performed by HCPs in ICU settings. Our study follows a wider variety of HCP roles than previous studies in order to examine in more depth how a system like the CCIS may impact the various roles differently. The CCIS introduction has included some types of bedside telemetry (patient monitors of vital signs) but not others (ventilators, for example), which may impact different HCP roles uniquely. Our study is ideally positioned to capture these myriad effects. Additionally, our study specifically examines staff tasks around shift change, at night, and on different days of the week. As informational continuity may be challenged during shift change [31], different factors may alter unit operations at night (e.g. different HCPs, different availability of staff), and on different days of the week (differing workloads based on numbers of operating rooms in use), this approach represents a more complete method of examining the effects of the CCIS on the ICU.

We have examined one perceived weakness of observational studies, participant reactivity. A second potential weakness surrounds the use of a 90-minute time limit for observations, which is aimed at limiting observer fatigue. At times, observers would perform two observations consecutively. We encouraged observers to take a short break in between observations to improve their alertness during each observation to help ensure high data quality. In fact, the literature supporting the use of a 90-minute time limit on observations is not entirely robust. We have been unable to find published findings supporting the use of a 90 minute time-limit, and it is very likely that this limit will vary from observer to observer. In the present study, we take a somewhat conservative approach in using a 90 minute limit, but we also allow observers the flexibility to complete up to three observations each day. In our experience, three daily observations can be completed accurately, as long as the observer takes longer breaks between each observation. The time of day that each observation is completed may also impact potential effects of observer fatigue. Multiple observations carried out during the middle of the night, for example, may be more likely to impact data quality. Our observers did not perform multiple observations at night.

A remaining question is the frequency with which interobserver reliability scores ought to be assessed. Although observers completed 'buddy' shifts alongside an experienced observer during training, there is some question in the literature as to how often observers should repeat these tests to ensure data quality. We ensure 85% agreement between observers before allowing trainees to complete their own observations. Data quality may be best determined by performing interobserver reliability scores among all pairs of observers. Depending on the number of observers, this task may or may not be feasible given resource constraints.

An additional potential weakness surrounds the use of the 'indirect patient care' category. The design of this category is such that activities like monitoring patient status, collecting medical equipment, and hand washing are included with activities based around information management such as reviewing patient charts and finding medical records. The time spent on these 'information tasks' may be important to investigate more closely as HCPs work with the CCIS to find the information that they need to provide patient care. If the process of finding and reviewing information is made more or less efficient for HCPs, the current indirect care category system would be unlikely to capture those changes. Effects involving time spent on reviewing documents may be diluted by other tasks in the 'indirect patient care' category. In addition, other categories could be enhanced to collect more detailed information about the tasks being performed, such as what information is being discussed during professional communication tasks. This consideration needs to be balanced with the possibility that any change that increases the complexity of the data definitions may make training the observers more difficult and could potentially impact the data quality. Additionally, significant changes to the data definitions may make comparison with other studies difficult or impossible. We would caution investigators that the work definitions they use at the outset of their studies may result in more or less valuable data depending on the aspects of HCP workflow they wish to examine.

The results showing time spent on documentation should be carefully interpreted due to the medication tasks category. The ICUs use medication orders as part of the patient charts. Unless an observer is standing much closer than the 3 metre following distance we employ, it may not be possible to identify every instance when medication orders are being written in the chart. If an observer stood closer to obtain more accurate records of medication related documentation, they would risk obstructing their participant. Thus, the values we have found may underreport medication prescribe and chart events, which may instead be captured in the documentation section. In general, we have sought to openly report potential weaknesses in this approach such that future investigators may benefit from this information.

## Conclusions

Canadian critical care providers spend greater proportions of time communicating with each other than do physicians and nurses working in Australian general hospital wards. This is consistent with specialized, coordinated, team based care. These results help to validate the previously published findings of the WOMBAT method on hospital wards as well as demonstrating the amounts of time front line critical care providers spend accessing and disseminating information for patient care. We describe a truly blended method with quantitative data resulting from subjective observations. Future studies will employ this method to examine how the CCIS impacts the time HCPs spend on their tasks, the interruptions they encounter, and whether the CCIS introduction is broadly a positive step for patient care.

## List of abbreviations

CCIS: Critical Care clinical Information System; ED: Emergency Department; GSICU: General System Intensive Care Unit; HCP: Health Care Provider; ICU: Intensive Care Unit; PDA: Personal Digital Assistant; PICU: Pediatric Intensive Care Unit; WOMBAT: Work Observation Method By Activity Timing

## Competing interests

The authors declare that they have no competing interests.

## Authors' contributions

MAB coordinated the observations, wrote the manuscript, and performed the data analysis. NTS is the senior author of the CCIS research study. She designed the study and provided editorial advice. DCM, RTNG, and JIW provided editorial advice. All authors provided direction and input into the study design, and reviewed and approved the final manuscript.

## Pre-publication history

The pre-publication history for this paper can be accessed here:

http://www.biomedcentral.com/1472-6947/11/32/prepub
